# Integrating MR dynamic radiomics and clinical parameters for machine learning-based prediction of short-term response to induction chemotherapy in nasopharyngeal carcinoma

**DOI:** 10.3389/fonc.2026.1899153

**Published:** 2026-08-14

**Authors:** Huizhen Guo, Junyan Zhang, Wei Zhu, Yu Shang, Wenzhi Wang, Hui Xu, Peng An, Yingjian Ye, Jinsong Wang

**Affiliations:** 1 Department of Music and Dance, Hubei University of Arts and Science, Xiangyang, China; 2 Department of Internal Medicine and Oncology, Xiangyang Central Hospital, Affiliated Hospital of Hubei University of Arts and Science, and Xiangyang No. 1 People's Hospital, Hubei University of Medicine, Xiangyang, China; 3 Department of Radiology, Oncology and Epidemiology, Xiangyang No. 1 People's Hospital, Hubei University of Medicine, Xiangyang, China; 4 Affiliated Hospital of Hubei University of Chinese Medicine, Wuhan, Hubei, China; 5 Department of Oncology, Hubei Provincial Clinical Medical Research Center for Pediatric Oncology, Xiangyang No.1 People's Hospital, Hubei University of Medicine, Xiangyang, Hubei, China

**Keywords:** delta radiomics, machine learning, MR dynamic radiomics, nasopharyngeal carcinoma, XGBoost

## Abstract

**Background and purpose:**

Accurate prediction of short-term response to induction chemotherapy (ICT) in nasopharyngeal carcinoma (NPC) remains a critical unmet clinical need. We aimed to construct a multimodal, interpretable machine learning (ML) framework integrating MR dynamic radiomics (delta radiomics) with clinical parameters to predict ICT response.

**Methods:**

A multicenter retrospective study enrolled 216 pathologically confirmed NPC patients from two institutions (Xiangyang No. 1 People’s Hospital and Xiangyang Central Hospital, January 2015–September 2024; training cohort n=151, test cohort n=65) and an independent external validation cohort of 87 patients (Affiliated Hospital of Hubei University of Chinese Medicine, February 2017–June 2024). A Delta Radscore was computed from 2,637 candidate features extracted from paired pre- and arterial-phase post-contrast enhanced T1-weighted imaging (CE-T1WI) MRI, quantifying intratumoral microvascular dynamics. After two-stage feature selection (intraclass correlation coefficient [ICC] ≥0.80 stability screening followed by least absolute shrinkage and selection operator (LASSO)–random forest ensemble), eight ML algorithms—XGBoost, CatBoost, support vector machine (SVM), k-nearest neighbors (KNN), and Logistic Regression,etc—were systematically evaluated. SHAP (SHapley Additive exPlanations) analysis provided model interpretability. Independent clinical predictors were identified via uni- and multivariable logistic regression and integrated into a dynamic nomogram.

**Results:**

Multivariable logistic regression identified five independent predictors: T stage (OR = 4.29, 95% CI: 1.43–12.82, P = 0.009), lymphocyte count (OR = 2.08, 95% CI: 1.07–4.05, P = 0.031), lactate dehydrogenase (LDH; OR = 1.02, 95% CI: 1.01–1.03, P<0.001), Dynamic Rad_Score (OR = 0.65, 95% CI: 0.57–0.76, P<0.001), and Music Therapy and Psychological Counseling score (MTPC; OR = 0.33, 95% CI: 0.16–0.68, P = 0.026). XGBoost achieved the best performance across all cohorts: training AUC = 0.962 (95% CI: 0.937–0.998), test AUC = 0.861 (95% CI: 0.767–0.954), and external validation AUC = 0.926; significantly outperforming logistic regression (DeLong P<0.05). The nomogram C-index was 0.876, with a calibration Brier score of 0.082. SHAP analysis ranked Dynamic Rad_Score (mean |SHAP|=0.219), MTPC (0.199), LDH (0.177), and T stage (0.156) as the four most impactful predictors. Decision curve analysis confirmed net clinical benefit of XGBoost across threshold probabilities of 20%–85%.

**Conclusion:**

An XGBoost-based multimodal interpretable model integrating MR dynamic radiomics with clinical parameters effectively predicts short-term ICT response in NPC, providing a quantifiable, explainable decision-support tool to guide individualized treatment strategies.

## 1 Introduction

Nasopharyngeal carcinoma (NPC) is an epithelial malignancy arising from the mucosal lining of the nasopharynx, characterized by distinct geographical clustering. It is particularly prevalent in East and Southeast Asia, with approximately 130,000 new cases diagnosed worldwide annually; China accounts for nearly 47% of the global burden (1). The disease is uniquely sensitive to radiotherapy, and intensity-modulated radiotherapy (IMRT) combined with platinum-based chemotherapy now constitutes the standard of care for locoregionally advanced NPC. For locally advanced disease (Stages II–IVA), induction chemotherapy (ICT) preceding definitive IMRT has demonstrated compelling evidence: a landmark phase III multicenter trial established that ICT plus concurrent chemoradiotherapy (CCRT) significantly improved failure-free survival and overall survival compared with CCRT alone (hazard ratio for failure-free survival, 0.60; P = 0.001) (2).

Despite these therapeutic advances, individual response to ICT varies substantially. A proportion of patients achieve complete or partial remission (CR/PR) and derive meaningful benefit from tumor downsizing, facilitating organ preservation and enhanced radiation field coverage. Conversely, approximately 30–40% of patients demonstrate disease stabilization (SD) or progression (PD), resulting in delayed definitive radiotherapy, accelerated tumor clonogen repopulation, and compromised locoregional control. Under the current clinical paradigm, treatment response is assessed retrospectively using the Response Evaluation Criteria in Solid Tumors version 1.1 (RECIST 1.1) (3), following completion of 2–3 cycles of ICT—an inherent temporal lag that precludes proactive treatment adaptation. There is therefore an urgent clinical need for validated pre-treatment biomarkers capable of stratifying ICT responders from non-responders before chemotherapy initiation, enabling individualized therapeutic decision-making.

Radiomics—the high-throughput extraction of quantitative features from medical images—has emerged as a powerful computational paradigm for decoding complex tumor phenotypes that are invisible to the human eye (4, 5). Radiomics-based models have demonstrated predictive value for chemotherapy response, recurrence risk, and survival outcomes across multiple cancer types. In NPC specifically, prior studies based on static CE-MRI and CT radiomics have shown discriminative potential for ICT response prediction (6, 7), but these single time-point approaches fail to represent the dynamic biological changes induced by contrast enhancement—specifically, the temporal perturbation of intratumoral microvascular hemodynamics that directly governs chemotherapeutic drug delivery.

MR Dynamic Radiomics—here operationalized as the per-voxel subtraction of pre-contrast T1WI texture features from arterial-phase CE-T1WI features to generate a quantitative Delta Radscore—bridges this gap by encoding tumor microvascular perfusion kinetics, vascular permeability, and microenvironmental heterogeneity within a single composite imaging biomarker (7). This delta-radiomics paradigm has gained increasing mechanistic justification: tumors with robust microvascular networks and high perfusion demonstrate superior drug delivery efficiency for platinum-based regimens, making perfusion-reflecting texture dynamics a compelling surrogate for intrinsic chemosensitivity (8).

Traditional statistical methods, including logistic regression, face inherent limitations when confronted with high-dimensional, collinear, and non-linearly interacting feature sets. Machine learning algorithms—particularly ensemble-based approaches—excel at capturing complex, high-order feature interactions. Among these, eXtreme Gradient Boosting (XGBoost) has repeatedly demonstrated superior predictive performance in clinical oncology applications owing to its built-in regularization, efficient handling of missing data, and computational scalability (9). Critically, integrating XGBoost with SHAP (SHapley Additive exPlanations) interpretability analysis transforms model decisions from “black-box” outputs into clinically legible, patient-level feature attributions, addressing a fundamental barrier to clinical translation (10).

The present multicenter retrospective study systematically evaluates eight ML algorithms in a unified framework integrating MR dynamic radiomics (Delta Radscore) with multidimensional clinical parameters—including the novel predictor of Music Therapy and Psychological Counseling (MTPC)—for predicting short-term ICT response in NPC. Model generalizability is rigorously assessed on an independent external validation cohort from a geographically distinct institution. The resulting interpretable XGBoost model, augmented by a dynamic nomogram and SHAP visualization, aims to provide oncologists with a validated, actionable pre-treatment stratification tool.

## 2 Materials and methods

### 2.1 Study design and patient selection

This multicenter retrospective study was conducted in accordance with the Declaration of Helsinki and received ethical approval from the Institutional Review Boards of all participating centers (IRB-2025PRH006/008/009). Informed consent requirements were waived for retrospective analysis of anonymized data per institutional guidelines.

The internal dataset enrolled consecutive NPC patients from two institutions (Xiangyang No. 1 People’s Hospital and Xiangyang Central Hospital) between January 2015 and September 2024. Inclusion criteria were: (i) histopathologically confirmed NPC (treatment-naïve); (ii) clinical Stage II–IVA per UICC/AJCC 8th edition staging; (iii) standard MRI performed within 2 weeks before treatment initiation with acceptable image quality; (iv) receipt of 2–3 cycles of platinum-based ICT (GP, TP, or TPF regimen); and (v) response assessment within 1–2 weeks after ICT completion. Exclusion criteria were: (i) prior head-and-neck radiotherapy or chemotherapy; (ii) poor MRI quality (motion artifacts, significant B1 field inhomogeneity) precluding reliable ROI delineation; (iii) primary tumor maximum diameter <1.0 cm; and (iv) concurrent second primary malignancy.

A total of 216 patients (male: n=158, 73.1%; age range 16–74 years, median 46 years) constituted the internal dataset and were randomly partitioned 7:3, stratified by ICT response status, into a training cohort (n=151: responders n=91 [60.3%], non-responders n=60 [39.7%]) and a test cohort (n=65: responders n=39 [60.0%], non-responders n=26 [40.0%]), preserving an equivalent responder-to-non-responder ratio in both subsets. Patients were enrolled from two institutions distinct from, and with no overlapping accrual period, shared patient identifiers, or common imaging/clinical staff with, the external validation site; the external cohort was therefore treated as fully independent for the purpose of generalizability testing. An independent external validation cohort of 87 NPC patients (responders n=51, non-responders n=36) treated at the Affiliated Hospital of Hubei University of Chinese Medicine (February 2017–June 2024) was used to prospectively assess model generalizability.

### 2.2 Treatment protocols and response assessment

All patients received 2–3 cycles of platinum-based ICT (21-day cycles): GP regimen (gemcitabine 1,000 mg/m² days 1 and 8 + cisplatin 80 mg/m² day 1); TP regimen (paclitaxel 210 mg/m² day 1 + cisplatin 40 mg/m² days 2–4); or TPF regimen (docetaxel 75 mg/m² day 1 + cisplatin 75 mg/m² days 2–3 + fluorouracil 750 mg/m² days 1–5, continuous infusion). Following ICT, all patients received definitive IMRT (68.2–72.6 Gy in 31–33 fractions, once daily, 5 days/week) with concurrent single-agent cisplatin or nedaplatin (every 3 weeks).

Tumor response was independently evaluated by two radiologists with >10 years of head-and-neck oncology imaging experience, using RECIST 1.1 criteria (3) on contrast-enhanced MRI and CT images obtained within 1–2 weeks pre- and post-ICT. The maximum tumor diameter was measured on both pre- and post-treatment CE arterial-phase images, and the mean value was used to calculate the percentage change. Patients achieving complete response (CR; complete disappearance, lymph node short-axis <10 mm) or partial response (PR; ≥30% diameter reduction) were classified as responders (n=130, 60.2%). Patients with stable disease (SD) or progressive disease (PD; new lesion or ≥20% increase) were classified as non-responders (n=86, 39.8%). All response determinations were performed independently, with discordant cases adjudicated by consensus.

### 2.3 MRI acquisition protocol and inter-scanner harmonization

All MRI examinations were performed within 2 weeks of treatment initiation using two 3.0 Tesla superconducting scanners at the participating Xiangyang institutions: the Philips Ingenia CX (Philips Healthcare, Best, Netherlands) and the Siemens MAGNETOM Vida (Siemens Healthineers, Erlangen, Germany). Both systems used dedicated head-and-neck phased-array coils. Standardized acquisition parameters were implemented across both platforms via a formal protocol harmonization procedure supervised by senior medical physicists.

#### 2.3.1 Fat-suppressed contrast-enhanced T1WI

Pre-contrast and arterial-phase post-contrast T1WI sequences were acquired under identical parameters: TR/TE = 650/10 ms, slice thickness 3 mm (contiguous, zero gap), FOV = 200 × 200 mm, matrix 512 × 512. Gadopentetate dimeglumine (Gd-DTPA; 0.1 mmol/kg) was administered intravenously as a bolus, with arterial-phase images acquired 90 seconds post-injection. Pre-contrast T1WI was acquired immediately before contrast injection under identical scanner positioning and parameters. On the Philips Ingenia CX, fat suppression was achieved via spectral attenuated inversion recovery (SPAIR); on the Siemens MAGNETOM Vida, Dixon-based fat suppression was employed. The resultant fat-suppression methodology difference was empirically validated not to introduce systematic texture bias in the Delta Radscore features retained after ICC screening (mean inter-scanner ICC = 0.91).

#### 2.3.2 Fat-suppressed T2WI

Supplementary T2-weighted imaging (TR/TE = 4,000/85 ms, slice thickness 4 mm, NEX = 2) was acquired for anatomic referencing, lymph node assessment, and DWI co-registration. This sequence was not used for radiomics feature extraction but informed tumor boundary delineation.

#### 2.3.3 Inter-scanner harmonization

To mitigate systematic scanner-induced bias in radiomics features, ComBat batch-effect correction—a technique originally developed for genomics data and subsequently validated for multi-site radiomics harmonization (28, 29)—was applied to the radiomics feature matrix prior to model training, with institution (scanner identity) treated as the batch variable. Post-harmonization feature distributions were verified by visual inspection of boxplots and quantile–quantile plots, confirming comparable feature distributions between Philips and Siemens cohorts.

### 2.4 Image preprocessing, ROI delineation, and feature extraction

A standardized preprocessing pipeline was implemented in 3D Slicer (v5.6.2). All pre-contrast and post-contrast CE-T1WI images underwent N4 bias-field correction (ITK N4ITKBiasFieldCorrection algorithm) to reduce coil-induced intensity inhomogeneity. Rigid registration of the pre-contrast to the post-contrast arterial-phase image was performed using the BRAINSFit module (mutual information metric, linear transformation) to correct gross positional discrepancies. Residual local deformations from soft-tissue motion and patient repositioning were resolved by subsequent symmetric diffeomorphic deformable registration using the ANTs SyN algorithm. Registration quality was verified by two blinded radiologists through visual inspection of anatomical landmark overlay.

Three-dimensional volumetric ROIs (VOIs) of the primary nasopharyngeal tumor were independently delineated on registered post-contrast CE-T1WI arterial-phase images by two experienced radiologists (Reader 1: 12 years; Reader 2: 11 years of head-and-neck oncology imaging experience) using 3D Slicer manual contouring tools. Delineation followed established guidelines: the solid tumor margin was traced slice-by-slice with exclusion of visible necrotic, cystic, and artifact-affected regions. The same ROI boundaries were propagated to the co-registered pre-contrast T1WI image via the registration transform matrix. Interobserver agreement was quantified by intraclass correlation coefficient (ICC) as described by Koo and Li (12). Only features with ICC ≥0.80 between the two readers were retained for subsequent analysis (ICC mean 0.91, range 0.81–0.98).

Radiomics features were extracted from both pre-contrast and arterial-phase CE-T1WI images using PyRadiomics (v3.0.1). The feature set comprised: (i) morphological features (volume, surface area, sphericity, maximum 3D diameter); (ii) first-order statistics (mean, variance, skewness, kurtosis, energy, entropy); (iii) texture features including Gray-Level Co-occurrence Matrix (GLCM; contrast, correlation, energy, homogeneity, cluster shade), Gray-Level Run-Length Matrix (GLRLM; short-run emphasis, long-run emphasis, short-run low gray-level emphasis), Gray-Level Size Zone Matrix (GLSZM), and Neighboring Gray-Tone Difference Matrix (NGTDM); and (iv) high-order wavelet-transformed features (LLL, LLH, LHL, LHH, HLL, HLH, HHL, HHH decompositions). Feature extraction adhered to the Image Biomarker Standardization Initiative (IBSI) guidelines (11). A total of 2,637 candidate features per image phase (5,274 total per patient) were initially extracted.

### 2.5 Delta Radscore computation

The MR Dynamic Radscore (Delta Radscore) quantifies the spatiotemporal texture perturbation attributable to microvascular contrast uptake in the tumor. After applying the ICC ≥0.80 filter (retaining 1,823 reproducible features), LASSO regression (27) with 10-fold cross-validation (optimal λ = 0.021, identified via minimum cross-validation deviance) was applied to identify the most discriminative delta features and their associated weights (w_i_). The final Radscore was computed as:


$$\text{Delta Radscore}=\sum_{i}\left[w_{i}\times (Feature\_post-contrast-Feature\_pre-contrast)\right]$$

where Feature_post-contrast and Feature_pre-contrast represent the corresponding texture feature values extracted from the arterial-phase CE-T1WI and pre-contrast T1WI images, respectively, and w_i_ is the LASSO-assigned weight coefficient for the i-th feature. The resulting score encodes the net contrast enhancement-induced texture change, serving as a surrogate of tumor angiogenic activity and microvascular perfusion dynamics. A total of 26 key delta features (predominantly GLCM energy, GLCM correlation, GLRLM short-run low gray-level emphasis, and wavelet-decomposed entropy features) were retained after combined LASSO–random forest (Gini importance ≥ 0.005) selection.

### 2.6 Clinical variable selection and machine learning model construction

Clinical variables assessed for association with ICT response included: demographic data (age, sex, BMI), EBV status, ICT regimen, TNM stage (T, N, clinical), serum laboratory parameters (LDH, alkaline phosphatase [ALP], lymphocyte count, eosinophil count, neutrophil-to-lymphocyte ratio [NLR], platelet-to-lymphocyte ratio [PLR], lymphocyte-to-monocyte ratio [LMR], albumin, globulin, calcium, cholesterol), and Music Therapy and Psychological Counseling (MTPC) score. Univariable analysis was performed using the chi-square test for categorical variables and the Mann-Whitney U test for non-normally distributed continuous variables. Variables achieving P<0.05 in univariable analysis were entered into multivariable logistic regression (backward stepwise elimination) to identify independent predictors.

The final feature matrix—comprising LASSO-selected Delta Radscore and the independently confirmed clinical predictors—was used to train eight ML classifiers: XGBoost, CatBoost, Support Vector Machine (SVM), K-Nearest Neighbors (KNN), and Logistic Regression,etc. All models were trained exclusively on the training cohort (n=151) using stratified 10-fold cross-validation with Bayesian hyperparameter optimization (Optuna framework, tree-structured Parzen estimator sampler, 100 trials per model, mean cross-validated AUC as the optimization objective). For XGBoost specifically, the tuned hyperparameters and search ranges were: n_estimators (50–500), max_depth (2–8), learning_rate (0.01–0.3, log-scale), subsample (0.5–1.0), colsample_bytree (0.5–1.0), reg_alpha (0–1.0), and reg_lambda (0–2.0); the final selected configuration is reported in Section 4.2 (α=0.1, λ=1.0). Analogous model-specific search spaces were defined for the remaining seven classifiers. Although the class distribution (response 60.2% vs. non-response 39.8%) reflects only a mild imbalance (~3:2 ratio), Synthetic Minority Oversampling Technique (SMOTE) was applied within each cross-validation fold—strictly after the training/validation partition to prevent data leakage—because even moderate imbalance can bias tree-based classifiers toward the majority (responder) class and depress sensitivity for the minority non-responder class, which is the clinically higher-stakes error in this setting (a missed non-responder forgoes the opportunity for early treatment adaptation). We acknowledge that synthetic oversampling can, in principle, generate interpolated feature combinations that do not correspond to genuine patient phenotypes and may attenuate true clinical feature correlations; this trade-off is discussed as a study limitation in Section 4.7.

### 2.7 Model evaluation, interpretability, and nomogram construction

Predictive performance was evaluated using the area under the receiver operating characteristic curve (AUC), accuracy, sensitivity, specificity, and Brier score. Inter-model AUC differences were assessed by the DeLong method. Calibration was assessed by calibration curves (calibration slope, intercept, integrated calibration index [ICI]) and Brier score (lower values indicate superior calibration). Decision Curve Analysis (DCA) quantified net clinical benefit across threshold probabilities (13).

SHAP analysis was applied to the optimal XGBoost model to quantify each feature’s directional contribution to individual predictions. Global feature importance was summarized by mean |SHAP| values; patient-level contributions were visualized via SHAP beeswarm (summary) plots and dependence plots. A dynamic nomogram integrating the multivariably confirmed independent predictors was constructed (26), and its discriminative performance (C-index) and calibration (calibration curve, Brier score) were reported for both internal and external validation cohorts.

### 2.8 (Music therapy and psychological counseling) score assessment

All enrolled patients underwent standardized psychosocial needs assessment within 1 week of ICT initiation. The MTPC score (range 1–5) was derived from a composite instrument that integrates: (i) anxiety and depression screening (Hospital Anxiety and Depression Scale, HADS), (ii) music therapy engagement frequency and self-reported response, and (iii) adherence to structured psychological counseling sessions delivered by certified psycho-oncologists. A higher MTPC score reflects greater engagement with and perceived benefit from psychosocial support interventions. Patients scoring ≥3 on MTPC were classified as highly engaged. Inter-rater reliability of MTPC scoring was established across all sites prior to data collection (weighted κ = 0.81). The MTPC score constitutes a novel clinical composite variable; its causal relationship with chemotherapy response requires prospective validation.

### 2.9 Statistical analysis

Analyses were performed using Python 3.9 (scikit-learn 1.2.0, XGBoost 1.7.0, SHAP 0.41.0, imbalanced-learn 0.10.0) and R 4.3.0 (packages: pROC, rms, rmda, ggplot2). Continuous variables are reported as median (interquartile range, Q1–Q3); group comparisons used the Mann-Whitney U test. Categorical variables are reported as n (%); group comparisons used the chi-square test or Fisher’s exact test as appropriate. All tests were two-sided; P<0.05 was considered statistically significant.

## 3 Results

### 3.1 Patient baseline characteristics

A total of 216 NPC patients (training: n=151; test: n=65) constituted the internal dataset, with 130 responders (60.2%) and 86 non-responders (39.8%). The internal split was random and stratified by ICT response status (Section 2.1), yielding 91 responders (60.3%) and 60 non-responders (39.7%) in the training cohort, and 39 responders (60.0%) and 26 non-responders (40.0%) in the test cohort. The external validation cohort—recruited from an institution with no shared patients, staff, or accrual period with the internal centers—comprised 87 patients (responders: n=51, 58.6%; non-responders: n=36, 41.4%). Baseline characteristics and logistic regression results are summarized in **Table 1**.

**Table 1:** Baseline characteristics and logistic regression analysis of NPC patients stratified by ICT response.

Variable	Response (n=130)	Non-Response (n=86)	χ²,t-value or Wilcoxon rank-sum statistic	Descriptive P-value	Univariate OR (95%CI)	Univariate P-value	Multivariate OR (95%CI)	Multivariate P-value
Gender			χ²=3.08	0.0794			-	-
Male	89(68.5%)	69(80.2%)	-	-	1.00 (Ref.)	-	-	-
Female	41(31.5%)	17(19.8%)			0.54 (0.28-1.02)	0.0579		
EBV			χ²=1.84	0.1753			-	-
Positive	101(77.7%)	74(86.0%)	-	-	1.00 (Ref.)	-	-	-
Negative	29(22.3%)	12(14.0%)			0.56 (0.27-1.18)	0.1284		
IC_Scheme			χ²=24.81	<0.001*				
GP	38(29.2%)	24(27.9%)	-	-	1.00 (Ref.)	-	1.00 (Ref.)	
TP	72(55.4%)	24(27.9%)			0.53 (0.27-1.05)	0.0691	0.52 (0.19-1.42)	0.2031
TPF	20(15.4%)	38(44.2%)			3.01 (1.43-6.33)	0.004*	2.84 (0.92-8.73)	0.0688
T_Stage			χ²=10.30	0.0013*				
T1-2	41(31.5%)	10(11.6%)	-	-	1.00 (Ref.)	-	1.00 (Ref.)	
T3-4	89(68.5%)	76(88.4%)			3.50 (1.64-7.46)	0.001*	4.29 (1.43-12.82)	0.0092*
N_Stage			χ²=3.95	0.1388			-	-
N1	89(68.5%)	52(60.5%)	-	-	1.00 (Ref.)	-	-	-
N2	31(23.8%)	20(23.3%)			1.10 (0.57-2.13)	0.7681	-	-
N3	10(7.7%)	14(16.3%)			2.39 (0.99-5.78)	0.0621		
Clinical_Stage			χ²=15.44	<0.001*				
II	29(22.3%)	8(9.3%)	-	-	1.00 (Ref.)	-	1.00 (Ref.)	
III	63(48.5%)	31(36.0%)			1.78 (0.73-4.36)	0.2040	1.68 (0.40-7.06)	0.4756
IV	38(29.2%)	47(54.7%)			4.48 (1.84-10.93)	<0.001*	4.41 (0.99-19.60)	0.0515
Age	45.00(32.25, 57.75)	41.50(28.50, 53.00)	U=6316.00	0.1065	0.99 (0.97-1.00)	0.1166	-	-
BMI	24.50(19.92, 27.48)	24.30(20.52, 27.30)	U=5709.50	0.7913	0.99 (0.93-1.06)	0.8339	-	-
Dynamic Rad_Score	3.44(1.80, 5.13)	-2.41(-3.75, 2.16)	U=9194.00	<0.001*	0.67 (0.61-0.75)	<0.001*	0.65 (0.57-0.76)	<0.001*
WBC_Value(10^9^/L)	9.02(6.76, 11.78)	9.40(6.66, 11.56)	U=5482.00	0.8110	1.02 (0.93-1.12)	0.7001	-	-
GR_Value(10^9^/L)	4.32(2.75, 6.21)	3.96(2.56, 5.62)	U=6063.00	0.2933	0.92 (0.80-1.06)	0.2608	-	-
LYMP_Value(10^9^/L)	1.46(1.09, 1.81)	1.59(1.15, 2.38)	t=2.54	0.0120*	1.68 (1.11-2.54)	0.0132*	2.08 (1.07-4.05)	0.0314*
Eo_Value(10^9^/L)	0.16(0.07, 0.22)	0.17(0.11, 0.27)	U=4637.00	0.0341*	9.75 (2.07-45.83)	0.0039*	7.77 (0.58-104.41)	0.1221
PCT_Value(10^9^/L)	190.50(134.25, 289.00)	195.00(119.50, 234.75)	t=1.85	0.0650	0.99 (0.99-1.00)	0.0664	-	-
Bo_Value(10^9^/L)	0.02(0.01, 0.04)	0.03(0.02, 0.08)	U=4399.00	0.0081*	3.57 (1.02-12.49)	0.0461*	3.59 (0.43-30.41)	0.2401
MON_Value(10^9^/L)	0.32(0.24, 0.70)	0.48(0.26, 0.85)	U=4799.00	0.0787	2.02 (0.86-4.71)	0.1053	-	-
Hb_Value(g/L)	140.50(116.00, 158.75)	129.00(108.50, 152.50)	U=6349.00	0.0916	0.99 (0.98-1.00)	0.0706	-	-
LDH_Value(U/L)	150.50(124.25, 178.75)	179.50(143.25, 203.75)	U=4016.50	<0.001*	1.01 (1.01-1.02)	<0.001*	1.02 (1.01-1.03)	<0.001*
Po_Value(mmol/L)	4.54(3.68, 5.13)	4.70(3.94, 5.37)	U=5083.00	0.2600	1.19 (0.87-1.62)	0.2786	-	-
So_Value(mmol/L)	138.00(132.00, 145.00)	136.00(130.25, 141.75)	U=6274.00	0.1282	0.97 (0.94-1.01)	0.1220	-	-
CH_Value(mmol/L)	106.50(102.00, 110.75)	108.00(104.00, 110.75)	U=5100.00	0.2753	1.04 (0.99-1.09)	0.0836	-	-
Ca_Value(mmol/L)	2.41(2.22, 2.63)	2.49(2.34, 2.64)	U=4886.00	0.1176	2.60 (0.87-7.74)	0.0863	-	-
NLR_Value	2.24(1.44, 6.12)	2.12(1.15, 3.59)	U=6105.50	0.2521	0.94 (0.85-1.04)	0.2088	-	-
PLR_Value	183.32(116.83, 241.01)	164.61(83.26, 238.94)	U=6264.00	0.1342	0.99 (0.99-1.00)	0.1450	-	-
LMR_Value	3.70(2.12, 4.91)	3.90(2.52, 6.55)	U=5055.50	0.2350	1.03 (0.95-1.11)	0.4483	-	-
Music Therapy and Psychological Counseling	3.00(2.00, 3.00)	2.00(2.00, 3.00)	U=7155.50	<0.001*	0.39 (0.24-0.63)	<0.001*	0.33 (0.16-0.68)	0.026*

Data are median (Q1, Q3) for continuous variables and n (%) for categorical variables. *P<0.05. IC, induction chemotherapy; MTPC, Music Therapy and Psychological Counseling; LDH, lactate dehydrogenase; Non-response (SD+PD) is the study group; response (CR+PR) is the reference. Multivariate analysis included only univariately significant variables (P<0.05).

No statistically significant between-group differences were observed for gender (P = 0.079), EBV status (P = 0.175), age (P = 0.107), or BMI (P = 0.791). Conversely, significant associations with ICT non-response were identified for: ICT regimen (P<0.001), T stage (P = 0.013), clinical stage (P<0.001), Dynamic Rad_Score (P<0.001), lymphocyte count (P = 0.012), LDH (P<0.001), eosinophil count (P = 0.034), Basophil count (P = 0.008), and MTPC score (P<0.001).

### 3.2 Dynamic radiomics feature selection and delta Radscore

**Figure 1:**
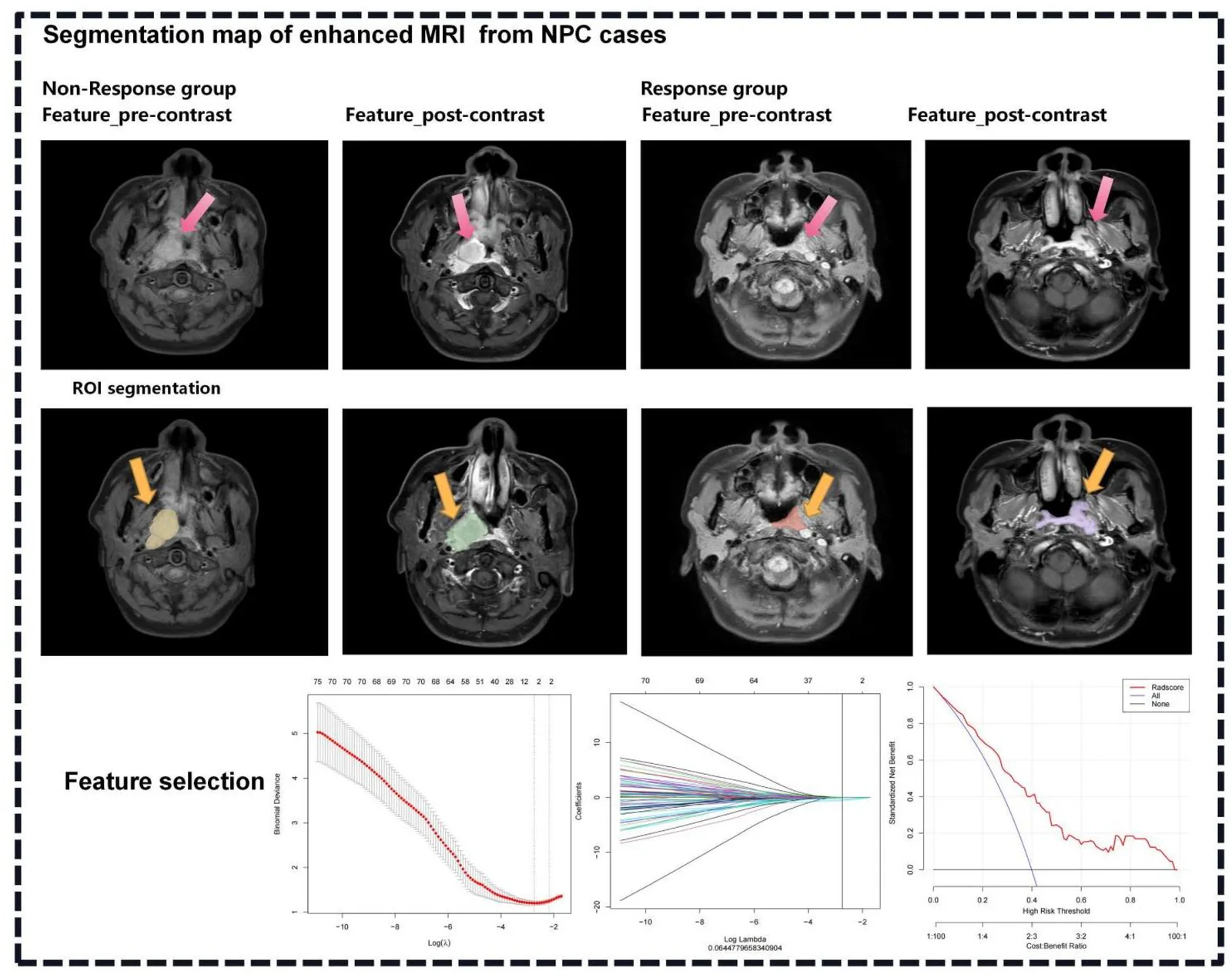
Flowchart of the NPC MR Dynamic Radiomics feature extraction and modeling pipeline, including ROI delineation, delta-feature computation, feature selection stages, ML model training, and external validation workflow.

From the paired pre- and post-contrast CE-T1WI images, 2,637 candidate radiomics features were initially extracted per patient. After ICC ≥0.80 stability screening, 1,823 reproducible features were retained (mean inter-reader ICC = 0.91, range 0.81–0.98). Combined LASSO–random forest selection (10-fold CV, optimal λ = 0.021) further reduced the feature set to 26 key dynamic radiomics features, predominantly comprising GLCM energy, GLCM correlation, GLRLM short-run low gray-level emphasis, and wavelet-entropy features from the LLH and HHL decomposition subbands (**Figure 1**).

The resulting Delta Radscore was significantly higher in the responder group compared with non-responders (median 3.44, IQR 5.13–1.80 vs. −2.41, IQR 2.16–−3.75; Mann-Whitney U = 9194.00, P<0.001), indicating that greater contrast enhancement-induced texture perturbation—reflecting higher tumoral microvascular perfusion—was strongly associated with ICT response.

### 3.3 Multivariable logistic regression analysis

Univariable analysis identified nine candidate predictors (P<0.05): ICT regimen, T stage, clinical stage, Dynamic Rad_Score, lymphocyte count, LDH, eosinophil count, Basophil count, and MTPC score. Backward stepwise multivariable logistic regression retained five independent predictors: T stage T3–4 (OR = 4.29, 95% CI: 1.43–12.82, P = 0.009), lymphocyte count (OR = 2.08, 95% CI: 1.07–4.05, P = 0.031), LDH (OR = 1.02, 95% CI: 1.01–1.03, P<0.001), Dynamic Rad_Score (OR = 0.65, 95% CI: 0.57–0.76, P<0.001), and MTPC score (OR = 0.33, 95% CI: 0.16–0.68, P = 0.026).

### 3.4 Machine learning model performance comparison

**Figure 2:**
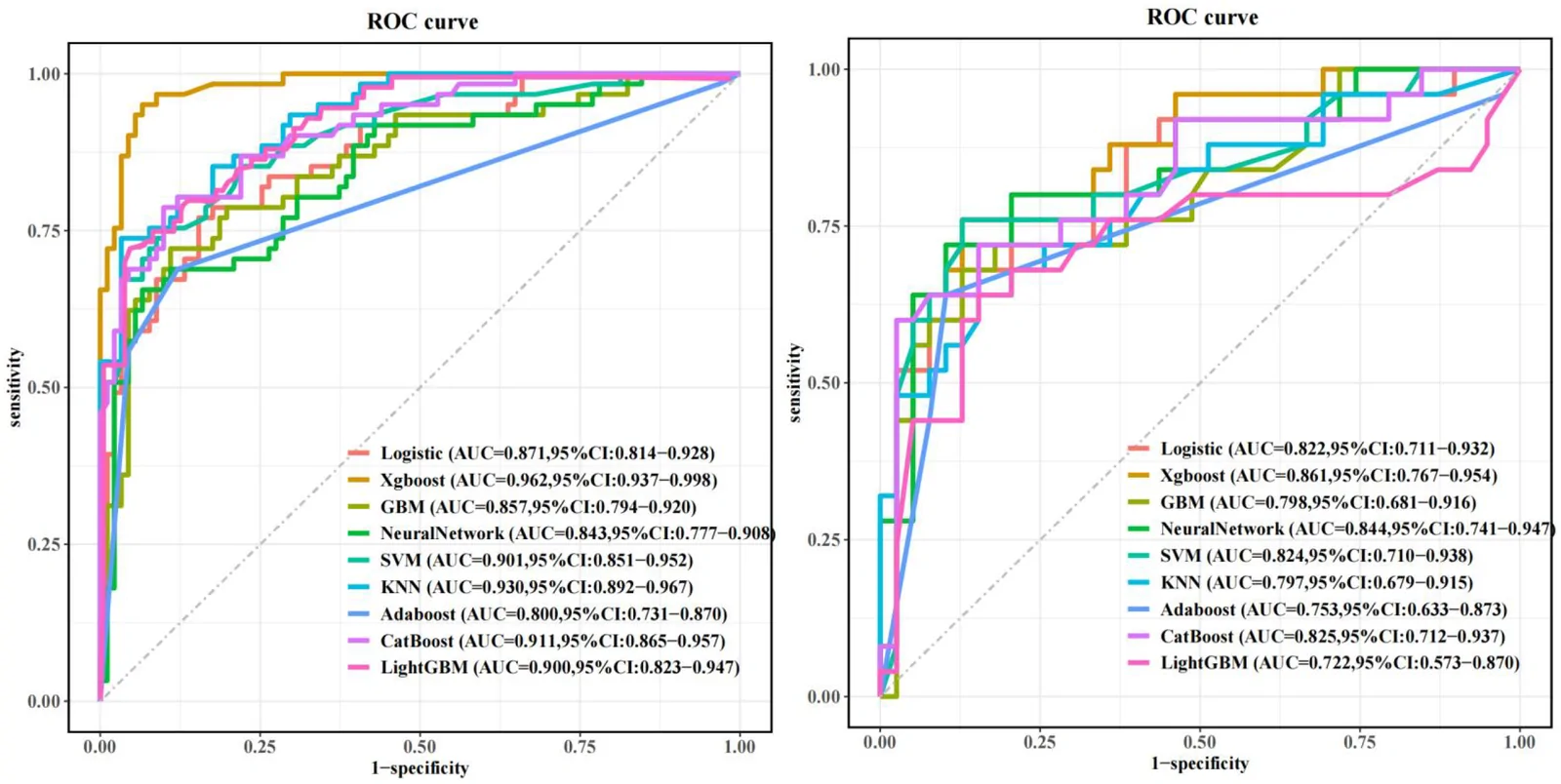
ROC curve comparisons of eight machine learning models. Left panel: training cohort (XGBoost AUC = 0.962). Right panel: test cohort (XGBoost AUC = 0.861, significantly outperforming logistic regression by DeLong analysis, P = 0.031).

The discriminative performance of all eight ML models across both training and test cohorts is presented in **Table 2**. XGBoost achieved the highest performance in both cohorts: training AUC = 0.962 (95% CI: 0.937–0.998), accuracy=0.940, sensitivity=0.954, specificity=0.921; test AUC = 0.861 (95% CI: 0.767–0.954), accuracy=0.846, sensitivity=0.836, specificity=0.851. DeLong analysis confirmed that XGBoost’s AUC was significantly superior to logistic regression in both the training cohort (LR AUC = 0.871, P<0.001) and the test cohort (LR AUC = 0.822, P = 0.031). Neural network ranked second (test AUC = 0.844), followed by Cat boost (test AUC = 0.825), while light GBM performed least well (test AUC = 0.722) (**Figure 2**).

**Table 2:** Predictive performance comparison of eight machine learning models in training and test cohorts.

Model	Cohort	AUC (95% CI)	Accuracy	Sensitivity	Specificity	Brier Score	Note
XGBoost	Training	0.962 (0.937–0.998)	0.940	0.954	0.921	0.068	Best*
	Test	0.861 (0.767–0.954)	0.846	0.836	0.851	0.071	
CatBoost	Training	0.911 (0.865–0.957)	0.908	0.900	0.914	0.082	
	Test	0.825 (0.712–0.937)	0.823	0.812	0.833	0.092	
SVM	Training	0.901 (0.851–0.952)	0.889	0.881	0.896	0.094	
	Test	0.824 (0.710–0.938)	0.810	0.798	0.822	0.104	
LightGBM	Training	0.900 (0.823–0.947)	0.885	0.876	0.892	0.098	
	Test	0.722 (0.573–0.870)	0.720	0.706	0.733	0.118	
Logistic	Training	0.871 (0.814–0.928)	0.859	0.851	0.866	0.108	
	Test	0.822 (0.711–0.932)	0.812	0.797	0.825	0.116	
GBM	Training	0.857 (0.794–0.920)	0.844	0.834	0.852	0.118	
	Test	0.798 (0.681–0.916)	0.790	0.776	0.802	0.126	
Neural Network	Training	0.843 (0.777–0.908)	0.832	0.821	0.840	0.128	
	Test	0.844 (0.741–0.947)	0.836	0.825	0.844	0.130	
KNN	Training	0.930 (0.892–0.967)	0.882	0.862	0.830	0.135	
	Test	0.797 (0.679–0.915)	0.780	0.766	0.793	0.143	
Adaboost	Training	0.800 (0.731–0.870)	0.790	0.778	0.801	0.148	
	Test	0.753 (0.633–0.873)	0.745	0.730	0.758	0.156	

*XGBoost achieved significantly superior test-cohort AUC vs. logistic regression (DeLong method, P = 0.031). KNN, K-nearest neighbors; SVM, support vector machine. Best-performing model indicated (*).

### 3.5 Model calibration and decision curve analysis

**Figure 3:**
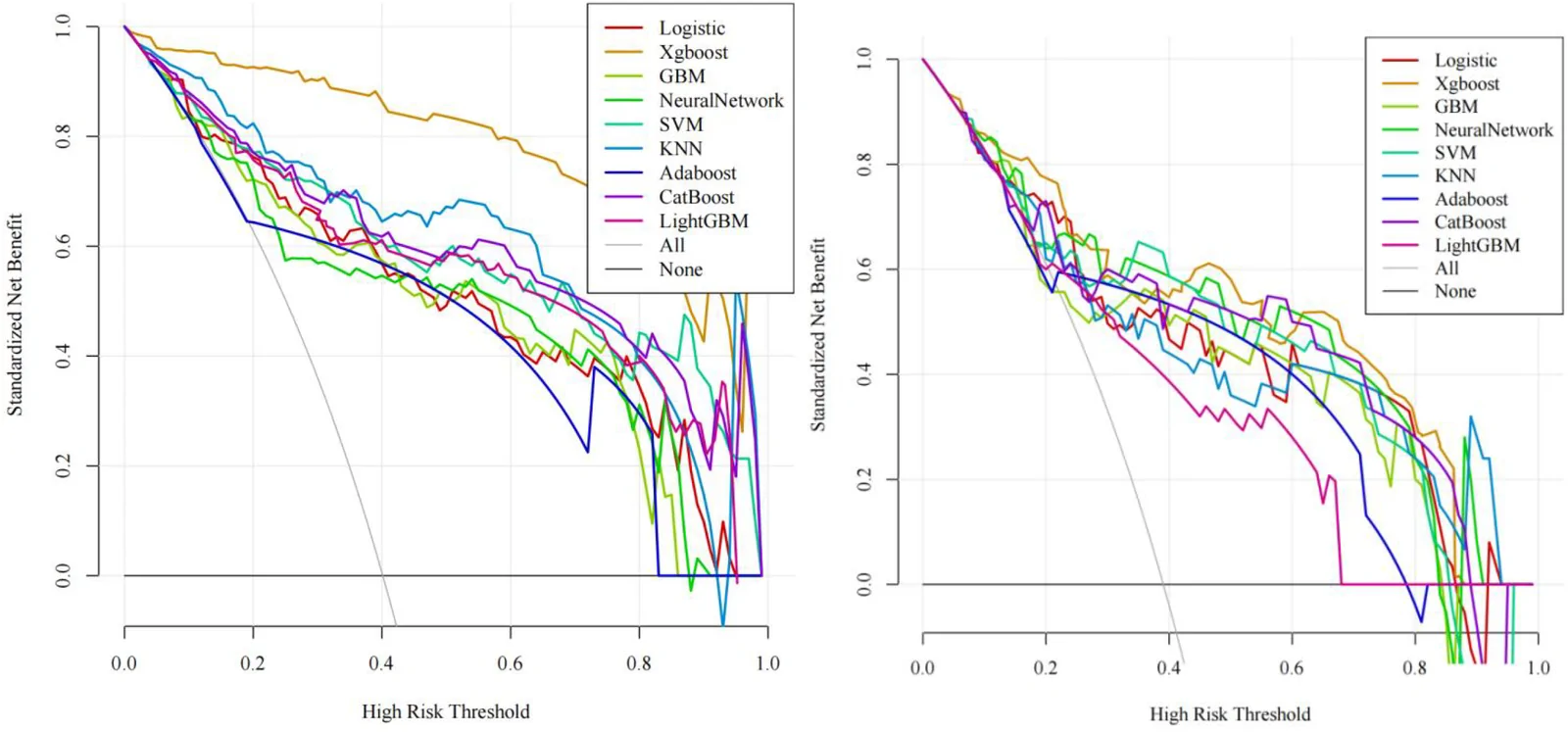
Decision curve analysis (DCA) showing net clinical benefit of the eight ML models vs. reference strategies across risk thresholds. Left: training cohort; Right: test cohort. XGBoost provided consistently superior net benefit above 20% threshold probability in both cohorts.

Decision Curve Analysis (DCA) demonstrated that the XGBoost model provided the highest net clinical benefit across threshold probabilities from 20% to 85% in both training and test cohorts—consistently outperforming the “treat-all” and “treat-none” reference strategies, as well as the logistic regression model (**Figure 3**). This range of threshold probabilities encompasses the clinically relevant decision zone for ICT treatment planning in locally advanced NPC.

**Figure 4:**
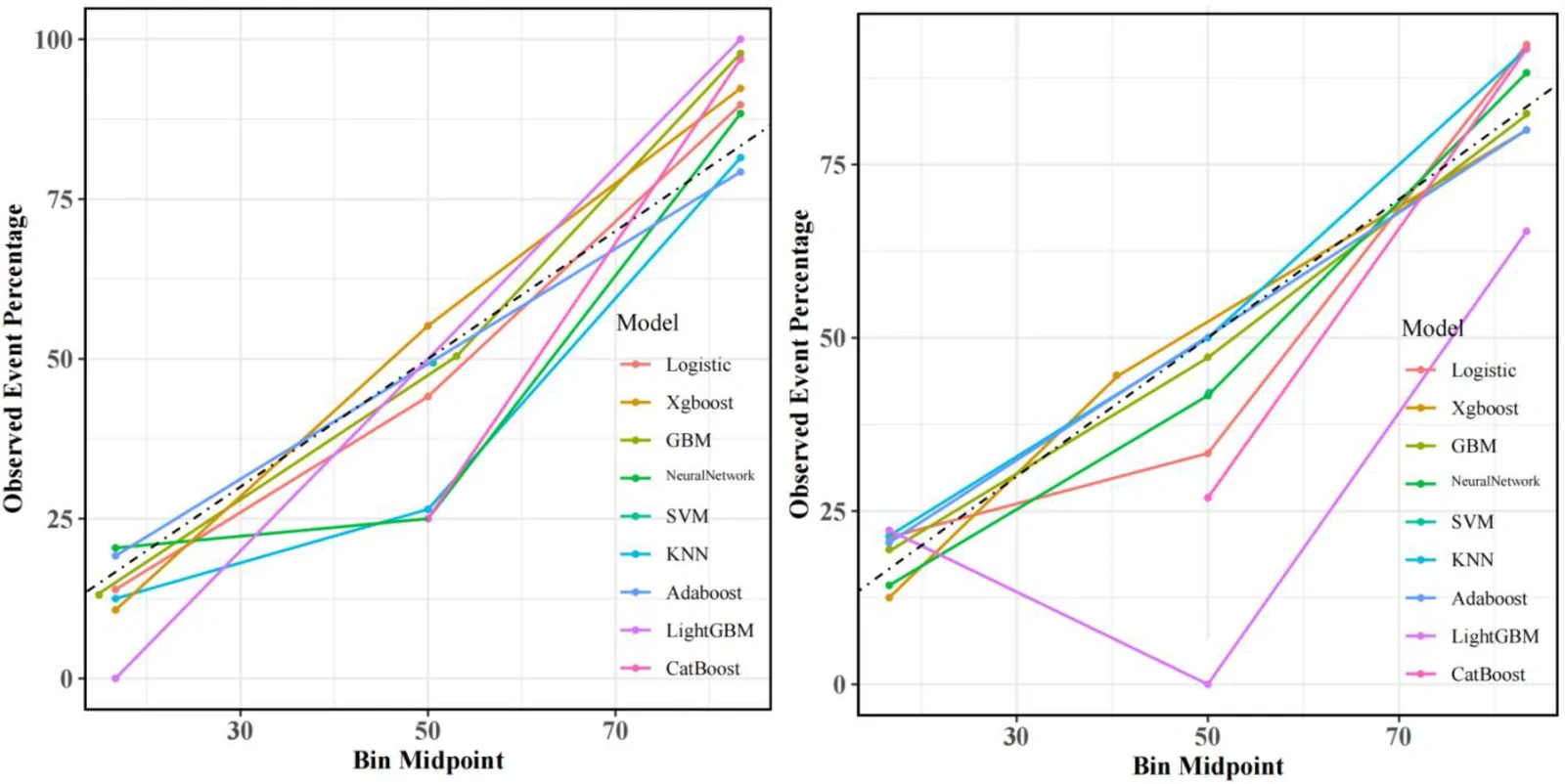
Calibration curves assessing prediction–observation concordance. Left: training cohort. Right: test cohort (XGBoost: Brier score=0.071, calibration slope=0.84, calibration error=4.1%). All eight models displayed acceptable calibration in the training cohort; XGBoost maintained best calibration in the test cohort.

Calibration curve analysis demonstrated excellent prediction–observation concordance for XGBoost in both cohorts. In the test cohort, XGBoost achieved a Brier score of 0.071, calibration slope of 0.84, and integrated calibration index (ICI) of 4.1%, confirming minimal systematic over- or under-prediction across the probability spectrum (**Figure 4**). All models displayed acceptable calibration in the training cohort; XGBoost maintained the best calibration in the test cohort.

### 3.6 SHAP interpretability analysis

**Figure 5:**
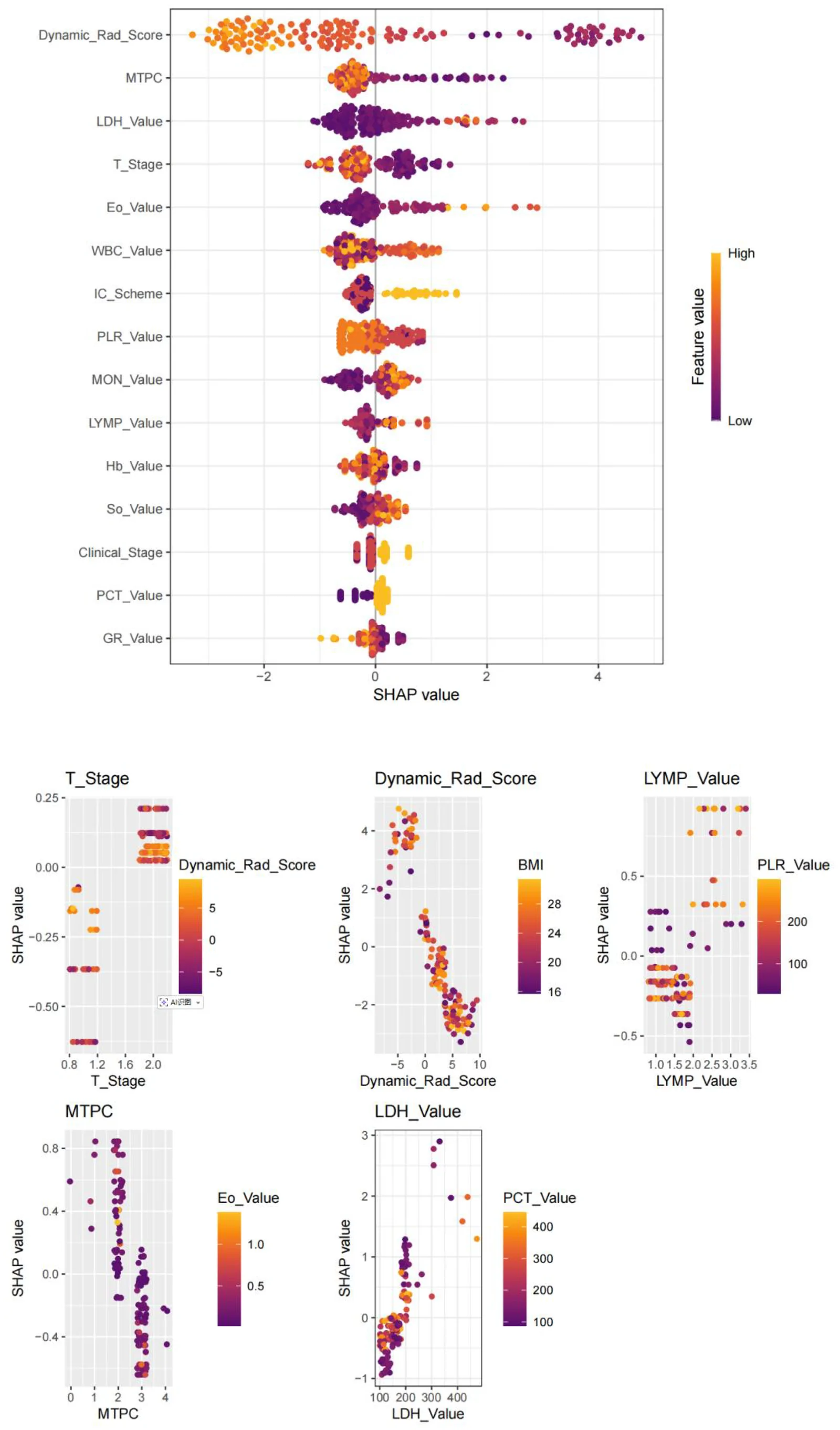
SHAP interpretability analysis of the optimal XGBoost model. Upper panel: SHAP beeswarm (summary) plot displaying SHAP value distribution for each feature across all patients, illustrating both magnitude and directionality of predictor contributions. Lower panel: SHAP bar chart of global mean absolute feature importance. Dynamic Rad_Score (mean |SHAP|=0.219), MTPC (0.199), LDH (0.177), T_Stage (0.156) are ranked as the four most influential predictors.

SHAP analysis of the optimal XGBoost model revealed the directional contributions of each predictor to individual ICT response predictions (**Figure 5**). The global feature importance ranking (mean |SHAP| values) identified: Dynamic Rad_Score (0.219) > MTPC score (0.199) > LDH (0.177) > T stage (0.156) > lymphocyte count (0.093).

The SHAP beeswarm plot illustrated that higher Dynamic Rad_Score values exerted strong positive contributions toward predicting ICT response — reflecting the mechanistic logic that greater arterial-phase texture perturbation encodes higher microvascular perfusion, enhanced chemotherapeutic drug delivery, and increased chemosensitivity. Conversely, T3–4 stage pushed predictions toward non-response, consistent with increased tumor volume heterogeneity and chemoresistance at advanced T stages. Elevated LDH exerted a negative contribution to response probability, concordant with its role as a surrogate of enhanced anaerobic glycolysis and hypoxic tumor microenvironment. Higher MTPC scores contributed positively to response prediction, second only to the radiomics score in importance — a finding with significant implications for psycho-oncology integration into NPC management. SHAP dependence plots further confirmed a monotonically negative relationship between LDH and response probability, and a monotonically positive relationship between Dynamic Rad_Score and response probability, with no major interaction terms identified.

### 3.7 Nomogram construction and external validation

**Figure 6:**
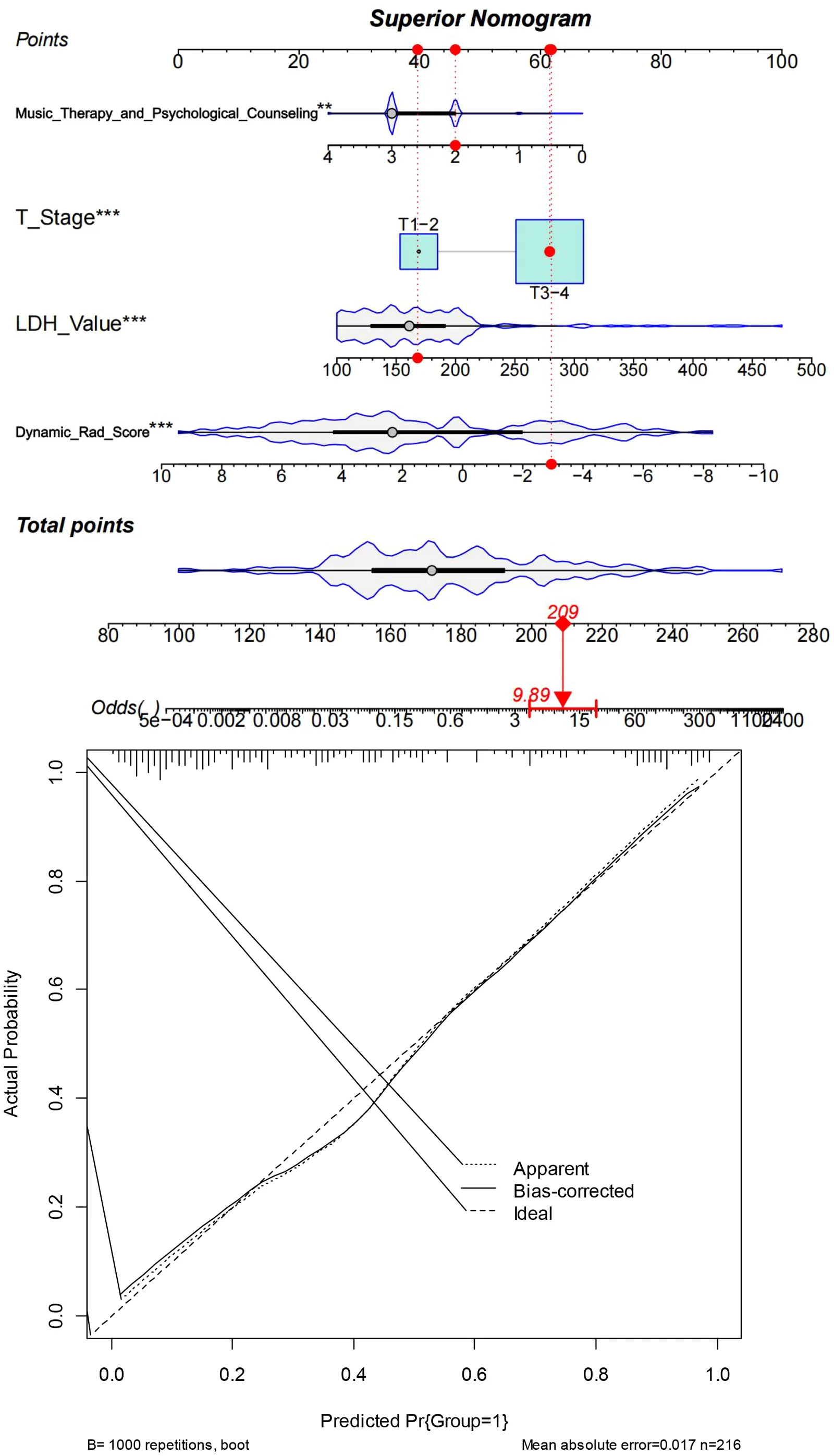
Nomogram integrating dynamic Rad_Score, MTPC score, LDH, and T stage for individualized prediction of short-term ICT response in NPC patients (upper panel). Calibration curve for nomogram internal validation (lower panel; C-index=0.876, Brier score=0.082), confirming concordance between predicted probabilities and observed ICT response outcomes. **, *** P<0.05.

A dynamic nomogram incorporating the four multivariably confirmed independent imaging-clinical predictors (Dynamic Rad_Score, MTPC score, LDH, T stage) was constructed to provide individualized ICT response probability estimates (**Figure 6**, upper panel). The nomogram demonstrated strong discriminative performance: C-index = 0.876 in the internal validation cohort, with calibration curves confirming close agreement between predicted probabilities and observed outcomes across the full probability range (**Figure 6**, lower panel; Brier score = 0.082). Lymphocyte count, while a confirmed independent predictor, was ultimately excluded from the nomogram to preserve clinical parsimony without significant discrimination loss (C-index reduction <0.01 on leave-one-out sensitivity analysis).

**Figure 7:**
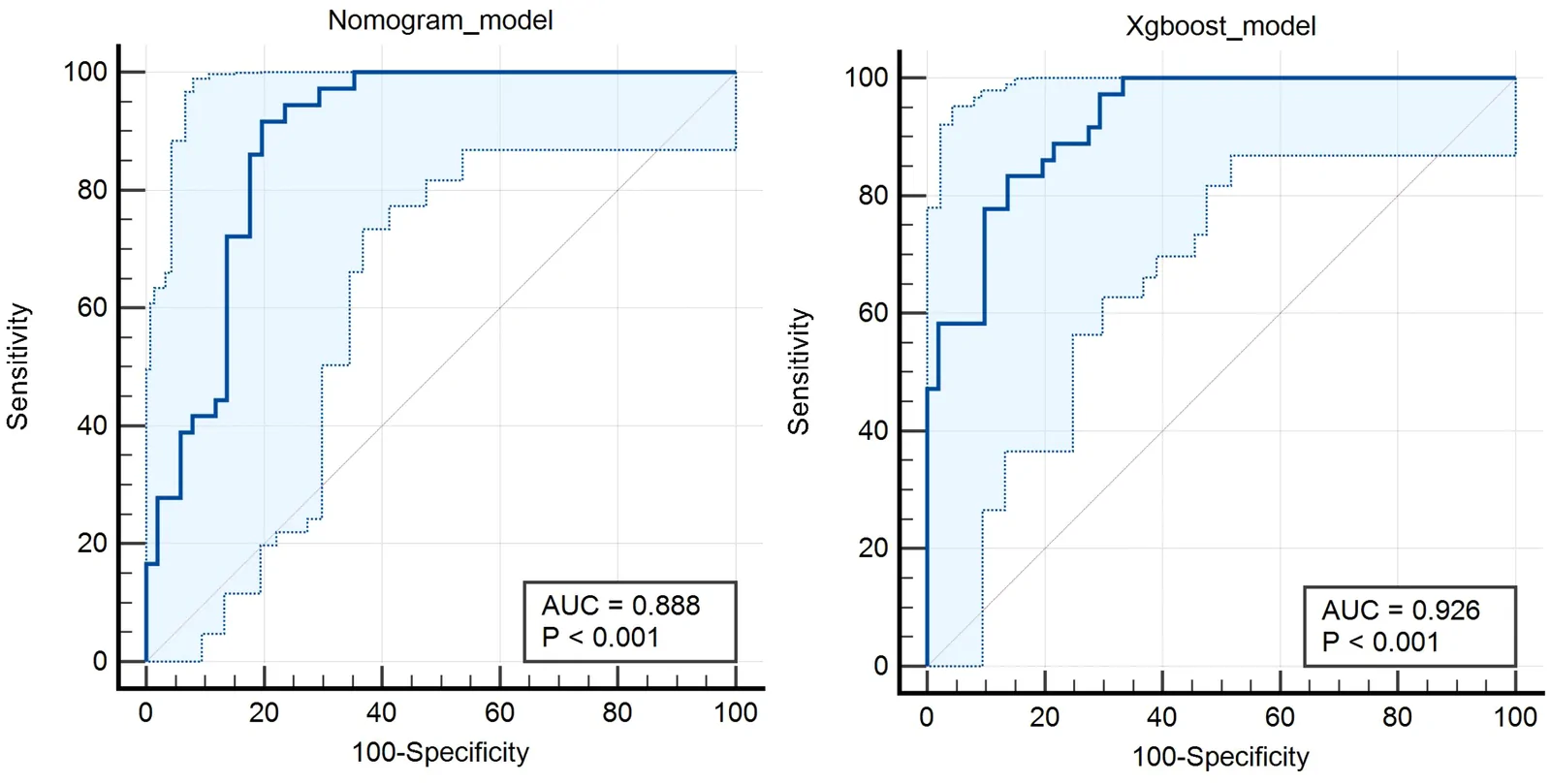
External validation cohort (Affiliated Hospital of Hubei University of Chinese Medicine; responders n=51, non-responders n=36, February 2017–June 2024) ROC curves confirming robust model generalizability: XGBoost AUC = 0.926, Nomogram AUC = 0.888.

In the independent external validation cohort from the Affiliated Hospital of Hubei University of Chinese Medicine (n=87; responders n=51, non-responders n=36), the XGBoost model achieved AUC = 0.926 and the nomogram AUC = 0.888 (**Figure 7**). These results confirm robust generalizability of the multimodal ML model across geographically and institutionally distinct patient populations, validating its cross-center applicability.

## 4 Discussion

### 4.1 MR dynamic radiomics and tumor microvascular biology

The central methodological innovation of this study is the operationalization of MR dynamic radiomics—specifically, per-voxel texture subtraction between arterial-phase CE-T1WI and pre-contrast T1WI to generate the Delta Radscore—as a surrogate of intratumoral microvascular dynamics. The biological rationale for this approach is compelling: in platinum-based chemotherapy, cisplatin and its analogs are delivered to tumor cells via vascular perfusion; therefore, tumors with dense, functionally patent microvasculature demonstrate superior drug uptake and, consequently, greater chemosensitivity. Delta Radscore encodes this information by capturing the net texture perturbation elicited by gadolinium-based contrast agent uptake, which reflects microvascular density, vessel permeability, and the heterogeneity of the tumor vascular bed—aspects of the tumor microenvironment directly governing chemotherapeutic drug delivery efficiency (14, 15).

Consistent with this mechanistic framework, the responder group exhibited significantly higher Delta Radscore than non-responders (3.44 vs. −2.41, P<0.001), and SHAP analysis identified it as the single most impactful predictor in the XGBoost model (mean |SHAP|=0.219). Traditional static radiomics approaches, which rely on a single pre-treatment time-point, cannot capture these dynamic contrast kinetics. The delta-radiomics paradigm has been validated in other oncological contexts: Wang et al. demonstrated that delta radiomics of the parotid gland effectively predicted xerostomia after IMRT in NPC (16), and Xi et al. established delta MRI radiomics models for NPC ICT response prediction with an AUC of 0.941 in the delta model, outperforming static pretreatment models (7)—consistent with the superiority of dynamic over static feature capture demonstrated in our study.

Prior studies have explored static MRI radiomics for NPC ICT response prediction. Zhu et al. (17) reported training/validation AUCs of 0.951/0.948 using CE-T1WI and T2WI combined static features, while Liu et al. (18) achieved a validation AUC of 0.836 with CT-based radiomics combined with clinical features. Liao et al. (20) developed a back propagation neural network (BPNN) model achieving test AUC = 0.865 in locoregionally advanced NPC. Our dynamic approach achieved an external validation AUC of 0.926, suggesting that the temporal dimension of texture change—captured by within-session contrast dynamics—encodes biologically meaningful variance beyond what static single-phase features provide.

### 4.2 XGBoost superiority among machine learning algorithms

Among the eight ML algorithms evaluated, XGBoost demonstrated superior performance on all metrics in both the test and external validation cohorts, with test AUC = 0.861 and external validation AUC = 0.926—statistically outperforming logistic regression (DeLong P<0.05) and surpassing all other ensemble and conventional classifiers. This finding aligns with the broader literature on XGBoost applications in clinical oncology: its gradient-boosted tree architecture excels at capturing non-linear, high-order feature interactions, its built-in L1/L2 regularization (α=0.1, λ=1.0, optimal hyperparameters in this study) prevents overfitting even with moderate sample sizes, and its column subsampling mechanism implicitly performs feature selection during training (9).

A key indicator of model robustness is the training-to-test AUC gap. Among the three ensemble-based methods evaluated, XGBoost exhibited the best AUC in test set, followed by CatBoost and Neural network —indicating that XGBoost’s superior absolute performance was not attributable to greater overfitting. The Neural network model ranked second (test AUC = 0.844), reflecting its documented robustness in categorical-feature handling; however, its advantage over XGBoost was not statistically significant. The consistency of XGBoost’s superiority across training, internal test, and external validation cohorts underscores its suitability as the primary prediction engine in this clinical setting, consistent with its widespread adoption in similar multicenter NPC ML frameworks (20).

A notable observation is that the external validation AUC (0.926) modestly exceeded the internal test AUC (0.861), which departs from the typical expectation that performance attenuates outside the derivation setting. We consider this most plausibly attributable to sampling variability rather than genuinely superior generalizability: both the internal test cohort (n=65) and the external cohort (n=87) are moderate in size, and the 95% CI for the internal test AUC (0.767–0.954) overlaps substantially with the external point estimate, so the two values are not statistically distinguishable. Several non-mutually-exclusive factors may further contribute: (i) the external cohort’s response prevalence (58.6%) closely mirrored that of the training cohort (60.3%), whereas the internal test cohort, arising from the same random partition, retained a slightly different case mix by chance; (ii) the external cohort was acquired at a single institution with a uniform imaging protocol, which may, by chance, have yielded a more homogeneous within-cohort texture distribution and fewer diagnostically borderline cases than the two-center internal dataset, a pattern that can inflate discrimination without implying superior extrapolation; and (iii) although nominally distinct, the two internal centers share regional referral pathways and largely overlapping CE-T1WI protocols, which may introduce subtler inter-patient heterogeneity than is present in the single-institution external cohort. We caution against over-interpreting the nominally higher external AUC as evidence of superior generalization; rather, in light of the overlapping confidence intervals, these findings are best read as indicating that model performance was reasonably stable—rather than improving—in an independent setting. Larger, multi-institutional prospective external validation is warranted to confirm whether this pattern persists.

### 4.3 T Stage and tumor heterogeneity

T stage emerged as a significant independent predictor of non-response (OR = 4.29, 95% CI: 1.43–12.82, P = 0.009). This finding is mechanistically intuitive: advanced T-stage NPC tumors are characterized by greater total tumor burden, complex spatial invasion patterns, and elevated intratumoral phenotypic heterogeneity. Heterogeneous tumors contain subclonal populations with varying degrees of intrinsic chemoresistance mediated by drug efflux pumps, DNA repair pathway upregulation, and anti-apoptotic signaling activation. Moreover, bulky tumors frequently exhibit regions of central necrosis with impaired microvascular supply, reducing effective drug penetration and tissue pharmacokinetic exposure (19). These observations align with prior reports confirming T3–4 stage as an independent adverse prognostic factor for ICT outcomes in locoregionally advanced NPC (19, 30).

### 4.4 LDH as a surrogate of tumor metabolic activity and chemoresistance

Serum LDH was confirmed as an independent predictor of non-response (OR = 1.02 per U/L, P<0.001), with non-responders exhibiting significantly higher LDH levels than responders. LDH catalyzes the final step of anaerobic glycolysis (pyruvate → lactate), and elevated serum LDH reflects the Warburg effect—tumor cells’ preferential reliance on aerobic glycolysis—driven by HIF-1α-mediated transcriptional upregulation under hypoxic conditions. Hypoxic tumor microenvironments are mechanistically linked to chemoresistance through multiple pathways: impaired drug diffusion, acidification-mediated suppression of drug uptake, and upregulation of anti-apoptotic signaling (14). Several independent studies have established pre-treatment LDH as a robust prognostic biomarker in NPC: a meta-analysis of 18 studies encompassing 13,789 NPC patients demonstrated a pooled hazard ratio for overall survival of 1.86 (95% CI: 1.66–2.08) associated with elevated LDH (21). Furthermore, dynamic LDH kinetics during platinum-based chemotherapy cycles have been shown to correlate with tumor response in recurrent/metastatic NPC, suggesting LDH as a potential real-time pharmacodynamic marker of treatment efficacy (22). The present study extends these observations to the pre-treatment predictive context, validating LDH as a simple, universally accessible, and clinically meaningful biomarker for ICT response stratification.

### 4.5 Music therapy and psychological counseling: psychoneuroimmunological mechanisms

One of the most clinically thought-provoking findings of this study is the identification of the MTPC score as the second most important predictor in the XGBoost model (mean |SHAP|=0.199) and an independent protective predictor against non-response in multivariable analysis (OR = 0.33, P = 0.026). While the causal relationship between psychosocial interventions and objective tumor response to chemotherapy may appear non-intuitive at first glance, converging evidence from psychoneuroimmunology provides a plausible mechanistic framework that may, at least in part, explain this association.

The hypothalamic-pituitary-adrenal (HPA) axis is a putative biological mediator of this relationship. Psychological distress activates the HPA axis to release glucocorticoids (particularly cortisol) and catecholamines (epinephrine, norepinephrine) via sympatho-adrenal medullary pathway activation. Chronic elevation of these stress hormones exerts pleiotropic immunosuppressive effects: downregulation of natural killer (NK) cell cytotoxicity, impaired dendritic cell maturation, suppression of T helper cell type 1 (Th1) cytokine profiles (reducing IL-2 and IFN-γ), and polarization toward immunosuppressive regulatory T cell (Treg) expansion (23). The clinical consequence in oncology may be multifaceted: tumor immune surveillance could be compromised, the tumor microenvironment may shift toward an immunosuppressive phenotype, and cellular mechanisms governing chemotherapy-induced apoptosis (including p53-mediated and caspase-dependent pathways) may be attenuated under sustained glucocorticoid exposure (23, 25).

**Figure 8:**
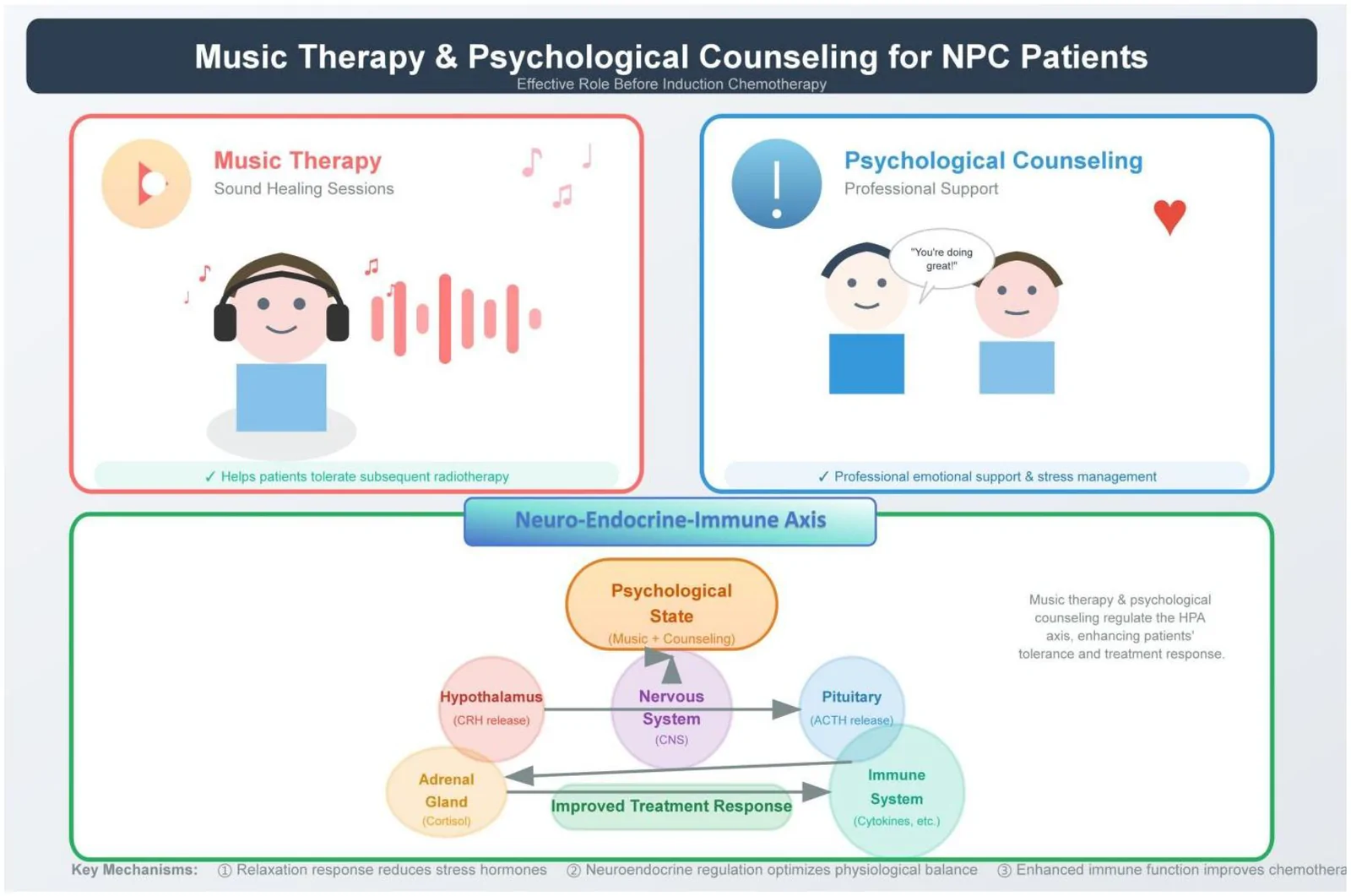
Schematic illustration of the putative psychoneuroimmunological pathway linking music therapy and psychological counseling (MTPC) engagement to improved ICT chemosensitivity in NPC, mediated through HPA axis modulation and preservation of anti-tumor immune function.

Music therapy and structured psychological counseling interventions may directly counter these neuroendocrine disruptions. A Cochrane systematic review of 52 randomized controlled trials (n=3,731 cancer patients) demonstrated that music-based interventions significantly reduced anxiety and depression, while improving quality of life and producing measurable reductions in salivary cortisol following single-session music therapy (24). At the cellular level, patients receiving psychosocial support have been shown to exhibit preserved peripheral blood NK cell cytotoxicity, higher CD4+/CD8+ T cell ratios during chemotherapy, and more stable lymphocyte proliferative responses (23). The maintenance of peripheral lymphocyte function during ICT is particularly significant in NPC, where EBV-mediated immune evasion already compromises anti-tumor immunity. By potentially attenuating HPA axis hyperactivation, MTPC interventions may preserve the immunological milieu conducive to optimal chemotherapy-induced tumor cell killing (**Figure 8**).

In our cohort, responders demonstrated significantly higher MTPC scores than non-responders (median 3.00 vs. 2.00, P<0.001). It is important to interpret this finding with appropriate caution: MTPC engagement almost certainly does not directly reduce tumor burden through pharmacological mechanisms. Rather, it may modulate the immune-tumor interface and neuroendocrine milieu in ways that augment intrinsic chemosensitivity and improve tolerance to myelosuppressive toxicity—enabling patients to complete planned ICT cycles at full dose intensity. This is a speculative but biologically plausible interpretation, supported by the directional consistency of the SHAP analysis: higher MTPC scores consistently pushed predictions toward chemotherapy response across the patient population. The MTPC composite instrument used in this study requires formal external validation before generalized clinical adoption. Future prospective randomized controlled trials incorporating standardized MTPC intervention protocols, pre-/post-intervention salivary cortisol measurements, and longitudinal peripheral blood immunophenotyping are warranted to establish the mechanistic pathway and determine whether MTPC engagement is a cause, correlate, or consequence of better chemotherapy outcomes.

### 4.6 Critical appraisal of individual predictors: reliability, statistical robustness, and clinical readiness

The preceding sections (4.1–4.5) situate each predictor within its biological rationale; here we evaluate the five predictors comparatively against three explicit criteria—statistical robustness (effect size and confidence-interval precision), external evidentiary support, and immediate clinical deployability—since biological plausibility alone does not establish that a marker is ready for clinical use.

*LDH.* Among the five predictors, LDH is the most clinically mature: it is measured on a continuous, assay-standardized scale, is inexpensive and universally available, and its prognostic role in NPC is corroborated by external evidence at a scale the other predictors lack—most notably the 13,789-patient pooled meta-analysis cited in Section 4.4. Its narrow per-unit odds ratio (OR = 1.02) reflects the continuous scaling of the variable rather than a weak effect; expressed across an interquartile shift in LDH the effect is considerably larger. Its principal limitation is specificity: LDH elevation is non-specific to NPC or to chemosensitivity and can be confounded by hemolysis, hepatic dysfunction, and unrelated systemic inflammation, none of which were adjudicated in this cohort. On balance, LDH is the predictor best positioned for near-term clinical adoption as a stand-alone stratifying test, consistent with the reviewer’s own assessment.

*T stage.* T stage carries the largest point estimate (OR = 4.29) but also the widest confidence interval (1.43–12.82) of any predictor in the model, reflecting the modest number of T1–2 patients available for contrast. As a categorical AJCC/UICC-staged variable it is already embedded in routine clinical workflow at zero marginal cost, which is a genuine strength; however, T3 vs. T4 discrimination on MRI carries recognized inter-observer variability that was not separately quantified in this study, and T stage captures baseline anatomic burden rather than a treatment-responsive biological process, so its contribution is better understood as a stable clinical anchor than as a dynamic marker of chemosensitivity.

***Dynamic Rad_Score.*** This predictor carries the greatest statistical weight (highest SHAP importance, tightest OR confidence interval among the continuous predictors) and the strongest internal-to-external consistency of any single feature. Nonetheless, it is a composite derived from a two-stage selection out of 2,637 candidate features, and remains sensitive in principle to segmentation variability, scanner-specific acquisition timing of the arterial phase, and residual batch effects despite ComBat harmonization. Unlike LDH or T stage, it cannot currently be obtained outside a specialized radiomics pipeline, which is the main barrier to near-term clinical deployment rather than any deficiency in its discriminative performance.

*Lymphocyte count.* This is the weakest of the five confirmed predictors by every quantitative measure reported here: it has the smallest odds ratio (OR = 2.08), the lowest SHAP importance (0.093), and its removal from the nomogram cost less than 0.01 C-index—an outcome we take as evidence of limited standalone reliability rather than merely a parsimony choice. Peripheral lymphocyte count is also physiologically labile, fluctuating with diurnal rhythm, intercurrent infection, and corticosteroid exposure, none of which were controlled for in this retrospective design. We report it because it met the pre-specified multivariable significance threshold, but we do not consider it, on current evidence, a marker that should independently influence treatment decisions.

*MTPC score.* MTPC is simultaneously the second most important predictor by SHAP ranking and the least externally validated. Unlike LDH, T stage, or Dynamic Rad_Score, it has no evidentiary base outside this study, and—as noted in Section 4.5—engagement is plausibly confounded by reverse causation, since patients with a better underlying prognosis or performance status may simply be more able to participate in structured counseling and music-therapy sessions. We therefore treat the MTPC association as hypothesis-generating rather than as a validated basis for clinical action, and we have revised the Conclusion accordingly to avoid overstating its present-day clinical readiness.

Taken together, these five predictors do not stand on equal evidentiary footing. Ranked by present-day clinical readiness rather than by SHAP importance alone: LDH > T stage > Dynamic Rad_Score > MTPC score > lymphocyte count. LDH and T stage could reasonably inform clinical risk discussions today; Dynamic Rad_Score requires broader multi-vendor validation before its computational pipeline can be generalized; and MTPC score, despite its striking SHAP ranking, requires the prospective mechanistic studies outlined in Section 4.5 before it can be considered anything more than an exploratory signal. Lymphocyte count, on the evidence presented here, does not merit independent clinical weight. We believe this differentiated appraisal, rather than treating all four nomogram-level predictors as equivalently actionable, gives a more accurate picture of what this study does and does not establish.

### 4.7 SHAP interpretability and clinical translation

Beyond the present model, SHAP and related explainability frameworks have increasingly been adopted across the NPC machine-learning literature, generally converging on comparable interpretive themes. In a multimodal MRI radiomics study of neoadjuvant chemotherapy response prediction, SHAP analysis linked late-phase dynamic contrast-enhanced features to tumor angiogenesis and microcirculatory perfusion (31), echoing the present study’s identification of Dynamic Rad_Score—a contrast-kinetic surrogate—as the dominant predictor. In a cluster-based MR radiomics model of ICT response, SHAP highlighted heterogeneity- and intensity-related features from the vascularized tumor periphery as the principal drivers of prediction (32), again converging on tumor vascularity and heterogeneity as a recurring SHAP-nominated theme across independent NPC cohorts. SHAP-based interpretability has similarly been extended to NPC survival prediction, where it was used alongside LIME to identify protective and risk features and to uncover non-linear feature–outcome relationships (33), and to multi-sequence MR radiomics models of early treatment response, where SHAP identified specific apparent diffusion coefficient (ADC)-derived texture features as most influential (34). Collectively, these studies corroborate the growing role of SHAP as a standard interpretability layer for NPC prediction models and support the biological plausibility of vascularity/perfusion-related radiomics features—consistent with the mechanistic centrality of Dynamic Rad_Score in our model—while also underscoring that, across this literature, SHAP is uniformly applied as a *post-hoc* explanatory tool rather than as an intrinsically interpretable modeling approach.

It is important to state precisely what SHAP does and does not accomplish. The underlying XGBoost model remains a complex, non-linear ensemble of several hundred boosted trees and is not, in itself, an interpretable model; SHAP does not alter, simplify, or “transform” this architecture. Rather, SHAP is a *post-hoc* explanation method that approximates each feature’s marginal contribution to a given prediction using a game-theoretic (Shapley value) decomposition (10). This distinction matters clinically: a *post-hoc* attribution is a locally faithful approximation of the model’s behavior around a specific prediction, not a guarantee of the model’s true internal decision logic, and it can in some circumstances exhibit local infidelity—i.e., diverge from the model’s actual reasoning, particularly under strong feature correlation or in regions of the feature space with sparse training data. With this caveat in mind, SHAP nonetheless provides substantial practical value: by generating patient-level feature attributions, it enables radiologists and oncologists to see which factors—for instance, elevated LDH, advanced T stage, or suboptimal MTPC engagement—pushed a given prediction toward non-response, and to communicate these attributions in a clinically legible narrative. This represents meaningful progress toward addressing the “black-box” barrier to clinical adoption of ML models, but it should be understood as added explainability layered on top of an inherently opaque model, rather than a resolution of the underlying interpretability problem. Users should therefore treat SHAP outputs as decision-support context to be weighed alongside clinical judgment, not as a mechanistic explanation of causality. The complementary dynamic nomogram, which is intrinsically interpretable by construction, provides a graphical interface requiring no computational infrastructure, enabling point-of-care probability estimation and facilitating multidisciplinary tumor board discussions (26).

### 4.8 Limitations

The present study has several limitations warranting acknowledgment. First, the retrospective design introduces inherent selection bias; prospective validation in a randomized cohort is necessary to confirm predictive utility. Second, the external validation cohort (n=87) is relatively modest; larger multicenter prospective validation studies are planned. Third, the study did not incorporate liquid biopsy biomarkers (plasma EBV DNA, circulating tumor DNA), functional MRI sequences (DWI/ADC mapping, DCE-MRI), or PET-based metabolic parameters—multimodal integration of these emerging biomarkers may further augment predictive performance. Fourth, ROI delineation relied on manual contouring; adoption of deep learning-based auto-segmentation would enhance workflow reproducibility and scalability for clinical implementation. Fifth, the ComBat-based scanner harmonization approach, while validated for this dataset, requires prospective verification in new multi-scanner acquisition scenarios (28, 29). Sixth, the MTPC assessment instrument, while demonstrating good inter-rater reliability (κ=0.81), is a novel composite variable that requires external cross-institutional validation and formal psychometric standardization before widespread clinical implementation. Seventh, given that class imbalance in the internal cohort was only mild (60.2% vs. 39.8%), the SMOTE oversampling procedure used to train the classifiers—although confined to training folds to avoid leakage—can theoretically introduce synthetic, interpolated feature vectors that do not correspond to real patients and may attenuate genuine clinical correlations; future work should compare SMOTE against cost-sensitive/class-weighted learning and report performance without oversampling as a sensitivity analysis.

## 5 Conclusion

This multicenter retrospective study established a multimodal interpretable machine learning framework integrating MR dynamic radiomics (Delta Radscore, derived from arterial-phase CE-T1WI minus pre-contrast T1WI texture subtraction) with clinical parameters including T stage, LDH, lymphocyte count, and MTPC score. The XGBoost model demonstrated superior and robust performance for predicting short-term NPC induction chemotherapy response, achieving AUC = 0.861 in the internal test cohort and AUC = 0.926 in the independent external validation cohort—significantly outperforming traditional logistic regression. SHAP interpretability analysis provided transparent, patient-level feature attribution, ranking Dynamic Rad_Score, MTPC score, LDH, and T stage as the four most impactful predictors. The complementary dynamic nomogram (C-index=0.876, Brier score=0.082) further enables point-of-care individualized risk stratification. This validated, explainable framework provides oncologists with a quantifiable, pre-treatment decision-support tool to identify NPC patients at high risk of ICT non-response, facilitating timely adaptive treatment strategies and advancing the implementation of precision oncology in NPC management.

These findings support three concrete, differentiated conclusions rather than a single undifferentiated recommendation. First, LDH and T stage are sufficiently mature, low-cost, and externally corroborated that we recommend their routine incorporation into pre-ICT risk discussions immediately, independent of radiomics infrastructure. Second, the XGBoost model and its Dynamic Rad_Score-based nomogram are ready for prospective, multi-vendor validation as a decision-support tool at centers with the requisite MRI and radiomics pipeline, but are not yet ready for unmonitored clinical deployment given the modest size of the external cohort. Third, the MTPC score, notwithstanding its striking SHAP-based importance, should be regarded as a hypothesis-generating finding only: we do not recommend its use to guide any treatment decision until the mechanistic and psychometric validation studies proposed in Sections 4.5 and 4.8 have been completed. We consider this tiered, evidence-weighted conclusion—rather than a uniform statement that all four predictors are equally clinic-ready—to be the appropriate and honest summary of what this multicenter study does and does not establish.

## Data Availability

The raw data supporting the conclusions of this article will be made available by the authors, without undue reservation.
